# CAPN2 correlates with insulin resistance states in PCOS as evidenced by multi-dataset analysis

**DOI:** 10.1186/s13048-024-01407-2

**Published:** 2024-04-12

**Authors:** Xi Luo, Yunhua Dong, Haishan Zheng, Xiaoting Zhou, Lujuan Rong, Xiaoping Liu, Yun Bai, Yunxiu Li, Ze Wu

**Affiliations:** 1 Faculty of Life science and Technology, Kunming University of Science and Technology, Kunming, China; 2 Medical school, Kunming University of Science and Technology, Kunming, China; 3 Department of Reproductive Medicine, NHC Key Laboratory of Healthy Birth and Birth Defect Prevention in Western China, the First People’s Hospital of Yunnan Province, Kunming, China; 4 Reproductive Medical Center of Yunnan Province, the Affiliated Hospital of Kunming University of Science and Technology, Kunming, China

**Keywords:** Polycystic ovary syndrome, Insulin resistance, Competing endogenous RNA network, Differentially expressed genes therapeutic drug prediction

## Abstract

**Objective:**

IR emerges as a feature in the pathophysiology of PCOS, precipitating ovulatory anomalies and endometrial dysfunctions that contribute to the infertility challenges characteristic of this condition. Despite its clinical significance, a consensus on the precise mechanisms by which IR exacerbates PCOS is still lacking. This study aims to harness bioinformatics tools to unearth key IR-associated genes in PCOS patients, providing a platform for future therapeutic research and potential intervention strategies.

**Methods:**

We retrieved 4 datasets detailing PCOS from the GEO, and sourced IRGs from the MSigDB. We applied WGCNA to identify gene modules linked to insulin resistance, utilizing IR scores as a phenotypic marker. Gene refinement was executed through the LASSO, SVM, and Boruta feature selection algorithms. qPCR was carried out on selected samples to confirm findings. We predicted both miRNA and lncRNA targets using the ENCORI database, which facilitated the construction of a ceRNA network. Lastly, a drug-target network was derived from the CTD.

**Results:**

Thirteen genes related to insulin resistance in PCOS were identified via WGCNA analysis. LASSO, SVM, and Boruta algorithms further isolated CAPN2 as a notably upregulated gene, corroborated by biological verification. The ceRNA network involving lncRNA XIST and hsa-miR-433-3p indicated a possible regulatory link with CAPN2, supported by ENCORI database. Drug prediction analysis uncovered seven pharmacological agents, most being significant regulators of the endocrine system, as potential candidates for addressing insulin resistance in PCOS.

**Conclusions:**

This study highlights the pivotal role of CAPN2 in insulin resistance within the context of PCOS, emphasizing its importance as both a critical biomarker and a potential therapeutic target. By identifying CAPN2, our research contributes to the expanding evidence surrounding the CAPN family, particularly CAPN10, in insulin resistance studies beyond PCOS. This work enriches our understanding of the mechanisms underlying insulin resistance, offering insights that bridge gaps in the current scientific landscape.

**Supplementary Information:**

The online version contains supplementary material available at 10.1186/s13048-024-01407-2.

## Introduction

PCOS is a prevalent endocrine disorder, affecting 6–10% of women of reproductive age worldwide [1], with regional variations in prevalence [2]. At its core, PCOS manifests through a triad of symptoms: hyperandrogenism, persistent anovulation, and polycystic ovaries, contributing to a wide range of reproductive and metabolic dysfunctions. These primary aspects underscore the complex pathogenesis of PCOS, which intertwines insulin resistance, chronic inflammation, genetic predispositions, and lifestyle factors such as obesity [3]. The condition poses significant challenges to reproductive health, notably increasing risks of infertility and menstrual irregularities. Moreover, PCOS patients frequently encounter comorbidities like metabolic syndrome, present in approximately 35.3% of cases, as well as non-alcoholic fatty liver disease and mental health issues, including anxiety and depression [4]. Current research emphasizes the critical role of lifestyle management and reproductive health awareness in PCOS, advocating for early detection and a comprehensive, multidisciplinary treatment approach [5–7].

Insulin resistance characterizes a state in which cells respond inadequately to insulin, playing a pivotal role in the pathophysiology of PCOS [8, 9]. Rather than being pathological, IR represents a challenging condition to quantify through conventional markers and is commonly regarded as a precursor to diabetes. In the context of PCOS, elevated insulin levels stemming from IR can further complicate metabolic issues, such as obesity and type 2 diabetes, and aggravate PCOS symptoms by promoting increased androgen production. Additionally, IR is associated with an elevated risk of cardiovascular diseases and metabolic syndrome among PCOS patients, underlining its significance in the broader spectrum of metabolic dysfunction [10, 11].

The management of insulin resistance in PCOS, though reliant on lifestyle interventions and medications like metformin [12], is significantly hampered by an insufficient understanding of the disease’s underlying mechanisms. This gap is particularly evident in the scarcity of detailed genetic studies, including gene expression profiles, which are crucial for elucidating the molecular basis of insulin resistance in PCOS [13]. The current shortfall in research into these mechanisms restricts the development of more effective and personalized treatment approaches. Advanced genetic research, especially focusing on the molecular intricacies of insulin resistance, is therefore imperative to overcome these limitations and enhance treatment efficacy in PCOS.

In this study, we embark on an innovative path by applying WGCNA and leveraging the MSigDB for a quantitative analysis of insulin resistance states. Our objective is to dissect the transcriptomic profiles of granulosa cells in PCOS patients, with a special focus on quantifying the insulin resistance status. This quantitative framework aims to facilitate the identification of critical hub genes that play a pivotal role in linking insulin resistance with PCOS. By mapping out the regulatory networks of noncoding RNAs associated with these central genes, we aspire to uncover new dimensions of the molecular mechanisms at play in PCOS. The ultimate goal is to illuminate the intricate genetic landscape governing insulin resistance within PCOS, thereby guiding the development of targeted and personalized treatment strategies. This endeavor contributes to the broader initiative of improving PCOS management through enhanced understanding of its underlying genetic factors.

## Materials and methods

### Data resource

For our investigation, multiple datasets from the past five years were carefully selected to investigate the molecular mechanisms of early localized insulin resistance status specifically in granulosa cells of PCOS patients. Datasets representing a wide range of body types within the PCOS population were included, and those with diagnosed insulin resistance were excluded. The goal was to identify characteristics of early-stage insulin resistance in PCOS. To ensure the accuracy of the study, datasets where participants had minimal differences in BMI were also excluded.

Ultimately, we chose four datasets from the Gene Expression Omnibus database (https://www.ncbi.nlm.nih.gov/geo/), each consisting of granulosa cell tissue samples from both PCOS patients and control subjects. It’s noteworthy that the diagnosis of PCOS in these datasets adhered strictly to the Rotterdam criteria [14]. Furthermore, control groups were meticulously matched with PCOS cases based on age to ensure the comparability of the study groups.

The primary dataset utilized was GSE80432 [15], analyzed with the Affymetrix Human Gene 1.0 ST Array (Platform GPL6244), comprising 16 samples split evenly between normal individuals and those diagnosed with PCOS. This dataset includes a wide array of body types, reflecting varying degrees of metabolic processes, which provides a comprehensive overview of the PCOS spectrum. Its diverse representation of PCOS variability makes it an optimal training set.

As a comprehensive validation dataset, GSE155489 [16] was employed, processed using the HiSeq X Ten platform (Platform GPL20795), which includes a total of 8 samples, evenly divided between normal and PCOS groups. Despite its smaller size, this dataset played a crucial role in further validating the diagnostic markers identified in the primary analysis.

To deepen our understanding of the regulatory mechanisms in PCOS, we constructed a ceRNA network, incorporating the GSE138518 lncRNA dataset [17] and the GSE138572 miRNA dataset [17], both analyzed on the Illumina HiSeq 2000 platform (Platform GPL11154). The former features 6 samples, equally divided between normal and PCOS subjects, while the latter comprises 10 samples, also evenly split between the two groups.

### WGCNA

The ssGSEA algorithm calculates IR scores for each sample by first ranking all genes according to their expression levels. It then assesses the relative position of IR-related genes within this ranked list to compute an enrichment score. This score quantifies the degree to which IR-related genes are overrepresented at the top of the ranked gene list, providing a numerical IR score for each sample. The approach allows for the direct quantification of IR pathway activity in individual samples based on gene expression data.

Furthermore, the gene set used for calculating IR scores was derived from 80 IR-related genes sourced from the MsigDB, specifically selected based on the ‘Insulin Resistance’ keyword within the HP_INSULIN_RESISTANCE pathway. MsigDB served as a crucial resource for our gene set enrichment analysis, facilitating an in-depth exploration of insulin resistance’s molecular basis in PCOS.

After quantifying IR scores, we proceeded to preprocess the normalized gene expression data. This preprocessing included the removal of genes that exhibited minimal variability across samples, defined by a MAD threshold of 0.1 or lower. Subsequently, hierarchical clustering was employed to identify outlier samples, resulting in the exclusion of sample GSM2127212 from our analysis. This methodical approach ensured the inclusion of only those genes displaying significant variability and samples that are representative of typical expression patterns.

With a clean dataset, we then proceeded to employ WGCNA to identify gene modules closely associated with IR. A soft threshold was determined to optimize the network topology, which is essential for constructing a meaningful gene co-expression network. We set the minimum size for each gene module at 70, ensuring a robust analysis.

The correlation between these gene modules and the quantified IR trait was subsequently calculated. This step enabled us to pinpoint key modules that exhibit a significant association with insulin resistance, underlining the modules’ potential role in the pathophysiology of PCOS related to IR. The integration of IR scores derived from ssGSEA with WGCNA highlighted the importance of a quantitative approach to understanding the genetic underpinnings of insulin resistance in PCOS patients.

### Differential expression genes analysis

In the analysis of the GSE80432 and GSE155489 datasets from the GEO, appropriate differential analysis methods were selected based on the characteristics of the downloaded data. For GSE80432, the limma package was utilized to assess differential expression between PCOS and normal samples within the mRNA expression matrix [18]. Limma, known for its robustness in small sample sizes and complex experimental designs, fits linear models and uses empirical Bayes methods for more precise variance estimates. In contrast, for GSE155489, the DESeq2 package [19] was employed, a method well-suited for analyzing count data from RNA sequencing experiments. This approach also involves fitting models to data but is specifically designed to handle the discrete count nature of sequencing data. For both datasets, the resulting *P*-values were used to identify significant differences between PCOS and normal samples, with a threshold of *P* < 0.05. Hub genes identified from WGCNA were then intersected with the DEGs from these datasets to identify a set of candidate genes. Enrichment analyses for these genes, including GO and KEGG, were conducted using the R software package ClusterProfiler [20], adhering to the same significance threshold.

### Machine learning refinement of identified DEGs

In the analysis of the GSE80432 dataset, feature dimensionality was reduced using the LASSO [21] logistic regression via R’s ‘glmnet’ package, focusing on selecting genes based on expression and grouping information for effective sample classification. Subsequently, key genes were ranked using the SVM [22] algorithm with RFE [23] through the ‘e1071’ package, assessing each gene’s importance and ranking based on error rate and accuracy. The Boruta method was then applied to further refine feature selection [24]. This algorithm employs Random Forest classification to iteratively compare actual features against randomly generated shadow features, effectively identifying the most significant ones. The final set of characteristic genes was determined by intersecting features identified by LASSO, SVM, and Boruta using the jVenn tool [25].

### Construction of ceRNA Network and Drug Target Prediction in IR-Related PCOS

To further investigate the role of key genes in insulin resistance-related PCOS, the study focused on elucidating the ceRNA regulatory network and identifying potential therapeutic targets. Differential expression analysis of lncRNAs and miRNAs was conducted on GSE138518 and GSE138572 datasets using the DESeq2 package. The analysis concentrated on mRNA-miRNA pairs with opposite regulation patterns, and lncRNA predictions were exclusively performed using the ENCORI database to select miRNA-lncRNA pairs demonstrating inverse regulation [26]. This led to the construction of a PCOS-specific ceRNA network based on the interactions of mRNA, miRNA, and lncRNA. The final phase involved identifying potential drug targets by querying each key gene against the CTD database (https://ctdbase.org/) [27], and the relationships between these drugs and key genes were visualized using Cytoscape software, forming a comprehensive drug-target network. This approach aims to advance the development of targeted therapies for IR-related PCOS.

### Specimen collection and qPCR procedures

Following approval from the Ethics Committee of the First People’s Hospital of Yunnan Province, granulosa cell tissues were obtained from patients diagnosed with PCOS. These patients were diagnosed based on the Rotterdam criteria, encompassing oligo- or anovulation, clinical and/or biochemical signs of hyperandrogenism, and the presence of polycystic ovaries. Patients with other endocrine disorders or gynecological conditions mimicking PCOS were excluded from the study. Informed consent was secured from all participants before tissue collection. The granulosa cells were harvested during routine oocyte retrieval procedures, typically part of IVF treatments, and were either immediately processed for RNA extraction or stored at -80 °C for subsequent analysis.

For qPCR analysis, total RNA was extracted from granulosa cell tissues using the TRIzol method (Thermo Fisher Scientific, Waltham, MA, USA). The integrity and concentration of the RNA were determined using the NanoDrop ND-1000 Spectrophotometer (Thermo Fisher Scientific, Waltham, MA, USA). For mRNA and lncRNA, the RNA was reverse-transcribed into cDNA using the Quantscript RT Kit (KR103, TIANGEN, Beijing, China). For miRNA analysis, cDNA synthesis was performed using the miRcute Plus miRNA First-Strand cDNA Kit (KR211, TIANGEN, Beijing, China).

qPCR was conducted on a Bio-Rad thermal cycler (CFX96 Touch, Hercules, CA, USA). The FastReal qPCR PreMix (SYBR Green, FP217, TIANGEN, Beijing, China) was used for mRNA/lncRNA analysis, and the miRcute Plus miRNA qPCR Kit (SYBR Green, FP411, TIANGEN, Beijing, China) for miRNA analysis. The qPCR conditions included an initial denaturation at 95 °C for 3 min, followed by 40 cycles of 95 °C for 15 s and 60 °C for 30 s. Expression levels of target genes and miRNAs were normalized to housekeeping gene GAPDH and internal control hsa-U6 (hsa-U6 qPCR Primer, CD201-0145, TIANGEN, Beijing, China), respectively, calculated using the 2^-ΔCt method. Primers for hsa-miRNA-433-3p (hsa-miR-433-3p qPCR Primer, CD201-0478, TIANGEN, Beijing, China) were used, and details of other primers are provided in Supplementary Table 1. All reactions were performed in triplicate to ensure accuracy and reproducibility.

### Analyzing the IR-Related Differential molecular markers in PCOS

Validation of bioinformatics findings on IR-related mRNA, lncRNA, and miRNA in PCOS was conducted using Prism 9 (GraphPad Software, San Diego, CA, USA). In this part of the analysis, qPCR data from PCOS patient samples were examined using non-paired t-tests, with a two-tailed *P*-value of < 0.05 indicating statistical significance. Additionally, the capacity of these markers to differentiate PCOS was assessed via ROC curve analysis, particularly through the calculation of the AUC. This process aimed to align empirical data with bioinformatics predictions, thereby confirming the roles of these markers in the context of PCOS.

## Results

### Identification of 3050 IR-Related hub genes in PCOS through WGCNA

**Fig. 1:**
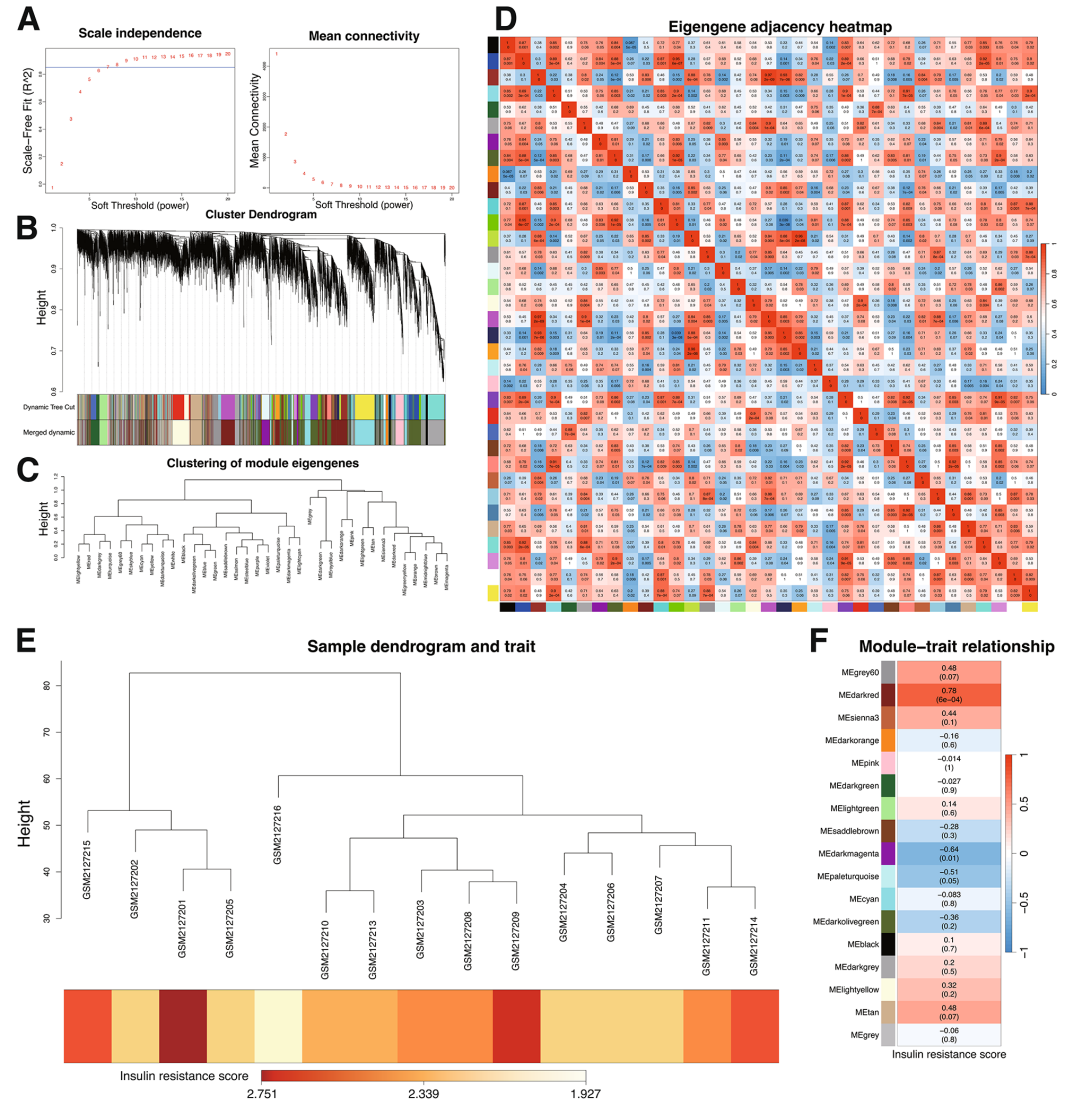
WGCNA Reveals Key Gene Modules Linked to Insulin Resistance in PCOS. (**A**) Plots scale-free fit index against mean connectivity to select the optimal soft-thresholding power, ensuring network accuracy and relevance. (**B**) The Module-Tree displays merging of similar modules at specific cut heights, simplifying the network. (**C**) Hierarchical clustering of gene modules is shown, with unique color names representing different modules, highlighting their relationships and potential functions. (**D**) An eigengene adjacency heatmap reveals correlations among module eigengenes, showing their network integration. (**E**) Combines sample clustering with a trait heatmap, linking gene module patterns to insulin resistance scores and identifying modules with notable correlations. (**F**) Details module-trait relationships with correlation coefficients and *P*-values, pinpointing modules significantly linked to insulin resistance, guiding focused therapeutic investigation

In the WGCNA (Fig. 1), a systematic analysis of over ten thousand genes initially resulted in 36 distinct modules, which were later refined to 17 key modules. This process was integral in mapping the complex genetic landscape associated with IR-related PCOS. The ‘darkred’ module, in particular, stood out for its strong correlation with IR score (|r| = 0.78), as determined by a rigorous threshold of *P* < 0.001. Comprising 3050 mRNAs, this module’s significant association highlights its potential impact on understanding the genetic underpinnings of IR-related PCOS.

### Thirteen essential genes identified in IR-related PCOS

**Fig. 2:**
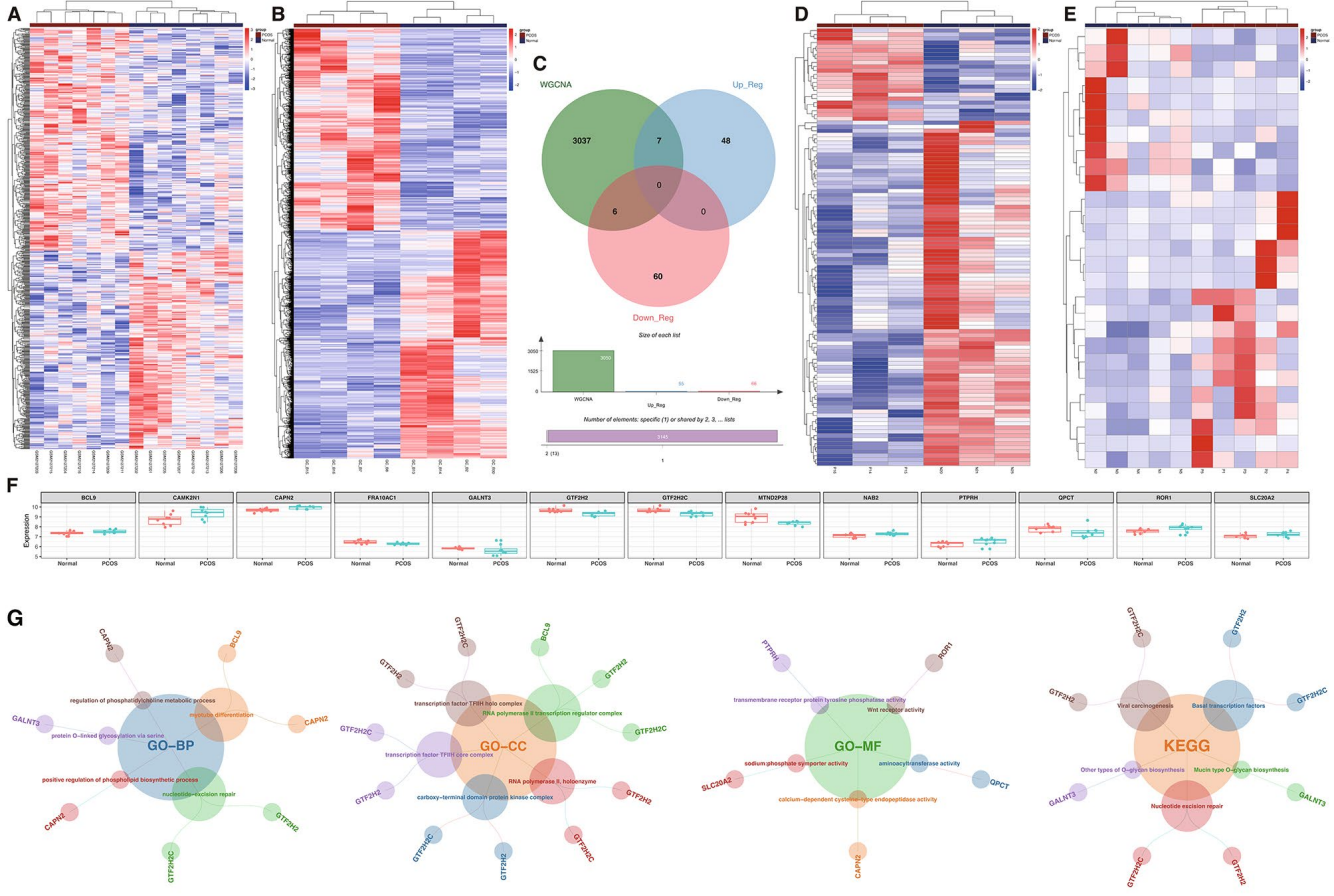
Integrated Analysis of Gene Expression with IR states in PCOS. (**A** & **B**) Display DEG heatmaps for datasets GSE80432 and GSE155489, highlighting differential expression between PCOS and controls, identifying potential biomarkers. (**C**) Features a Venn diagram merging “darkred” module genes from WGCNA with DEGs, isolating key genes linked to IR in PCOS. (**D** & **E**) Present expression heatmaps for lncRNAs and miRNAs in GSE138518 and GSE138572, revealing regulatory non-coding RNA changes in PCOS. (**F**) Box plots of 13 genes showcase distinct expression levels in PCOS versus controls, pinpointing IR-related genes. (**G**) Offers GO and KEGG pathway analyses, elucidating the biological impact of these genes in PCOS.

Differential gene analysis in the GSE80432 and GSE155489 datasets was performed to identify variations in genes between disease and normal sample groups. In GSE80432, 723 DEGs were identified, while in GSE155489, there were 5116 DEGs. Heatmaps were used for clustering analysis of these DEGs, illustrated in Fig. 2A and B.

In WGCNA, the most significant module, labeled “darkred,” contained 3050 DEGs. The analysis first involved intersecting the upregulated DEGs from GSE80432 with those from GSE155489, followed by a similar intersection for downregulated DEGs. These groups of intersected upregulated and downregulated DEGs were then each compared separately with the genes from the “darkred” module in WGCNA (Fig. 2C). This stepwise approach was critical to identify relevant gene overlaps.

This process identified 13 key DEGs (Fig. 2F), with 7 upregulated (BCL9, CAMK2N1, CAPN2, NAB2, PTPRH, ROR1, SLC20A2) and 6 downregulated (FRA10AC1, GALNT3, GTF2H2, GTF2H2C, MTND2P28, QPCT).

Enrichment analyses for these 13 IR-related significant DEGs were conducted using KEGG and GO. This revealed 5 KEGG pathways and 125 GO pathways, including 78 BPs, 27 CCs, and 20 MFs, detailed in supplementary Table 2. The top 5 pathways in both KEGG and GO were showcased in Fig. 2G. Notably, CAPN2 was primarily involved in BP processes like the regulation of phosphatidylcholine metabolic process, myotube differentiation, and positive regulation of phospholipid biosynthetic process, as well as MF processes such as calcium-dependent cysteine-type endopeptidase activity.

### Pinpointing CAPN2 in IR-related PCOS through LASSO, RFE-SVM and Boruta analysis

In the LASSO regression model, applied to the GSE80432 dataset and focusing on the 13 key genes identified from the differential gene expression analysis, the regression was conducted using R’s ‘glmnet’ package with standard parameters. This setup facilitated LASSO logistic regression, enabling the calculation of error rates for different features during cross-validation. The results were illustrated through a gene coefficient graph and a cross-validation error plot. The analysis identified a lambda.1se value of 0.117 as the point of minimum error rate, leading to the selection of the following feature mRNAs: CAPN2, CAMK2N1, GTF2H2, and GTF2H2C.

The SVM algorithm, implemented through the R package “e1071,” was utilized to rank the 13 key mRNAs. The RFE method was employed to iteratively refine the selection, focusing on the importance and ranking of each mRNA. This process involved calculating the error rate and accuracy for each iteration, with the optimal combination of mRNAs selected based on the lowest error rate. The analysis demonstrated peak accuracy and lowest error with the selection of the top 1 feature, CAPN2.

To further ensure the robustness of results, the Boruta algorithm was employed as an additional validation step. This method confirmed the relevance of several genes previously identified, solidifying their roles in IR-related PCOS. The Boruta analysis resulted in the confirmation of 8 key genes: CAPN2, PTPRH, SLC20A2, ROR1, GTF2H2, GTF2H2C, QPCT, and GALNT3, while 6 genes were deemed less significant and categorized as rejected.

**Fig. 3:**
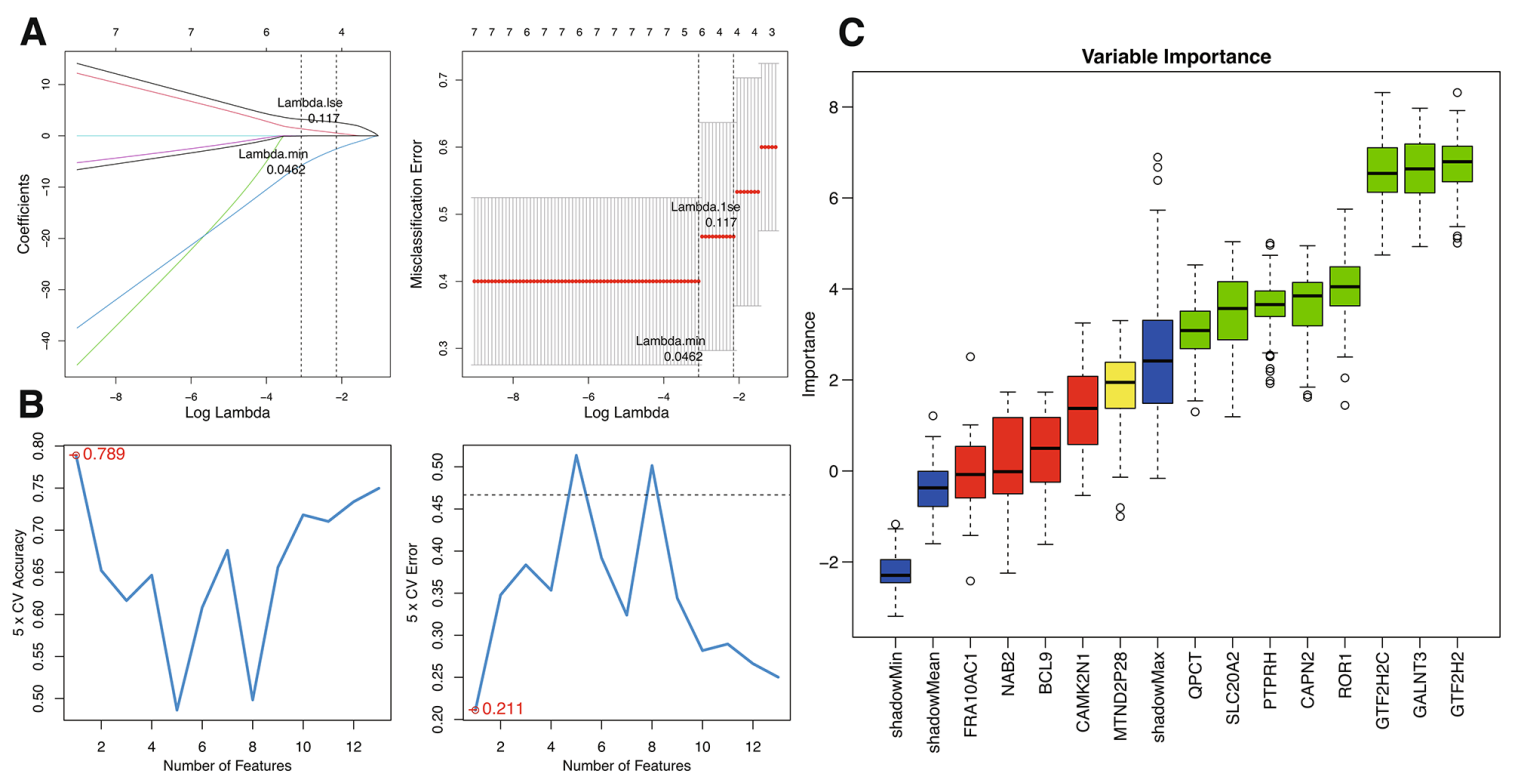
Machine Learning for Pivotal Gene Selection. (**A**) LASSO regression is shown via coefficient trajectories against log(lambda) on the left, identifying optimal gene selection parameters: ‘Lambda.min’ for minimal error and ‘Lambda.1se’ for a simpler model. On the right, a misclassification error plot indicates the best-performing model against lambda values for robust PCOS gene selection. (**B**) The RFE analysis correlates the number of features with model accuracy (left) and cross-validation error (right). The plots highlight the optimal number of predictive features that correspond to the highest accuracy and lowest error, optimizing the gene selection for PCOS. (**C**) Boruta’s boxplot assesses gene importance against shadow features to confirm the significance of genes in PCOS, distinguishing those with substantial contributions to the pathogenesis of IR.

The methodologies employed in this study demonstrate robustness in identifying key mRNAs associated with IR in PCOS (Fig. 3). The intersected results reinforced the significance of these methodologies. Notably, this intersection pinpointed CAPN2 as the singular, crucial gene consistently selected across various analytical methods, affirming its pivotal role in IR-related PCOS.

### ceRNA Network Construction and Drug Target Prediction in IR-Related PCOS

In this study, we conducted a differential expression analysis of lncRNAs and miRNAs in PCOS and normal samples, using the DESeq2 package in R, applied to datasets GSE138518 and GSE138572. Specifically, we identified 109 significantly different lncRNAs in GSE138518 and 27 miRNAs in GSE138572, when comparing PCOS to normal samples. Cluster analysis was performed to visualize the distribution of these differentially expressed lncRNAs and miRNAs, as shown in a heatmap (Fig. 2D and E).

**Fig. 4:**
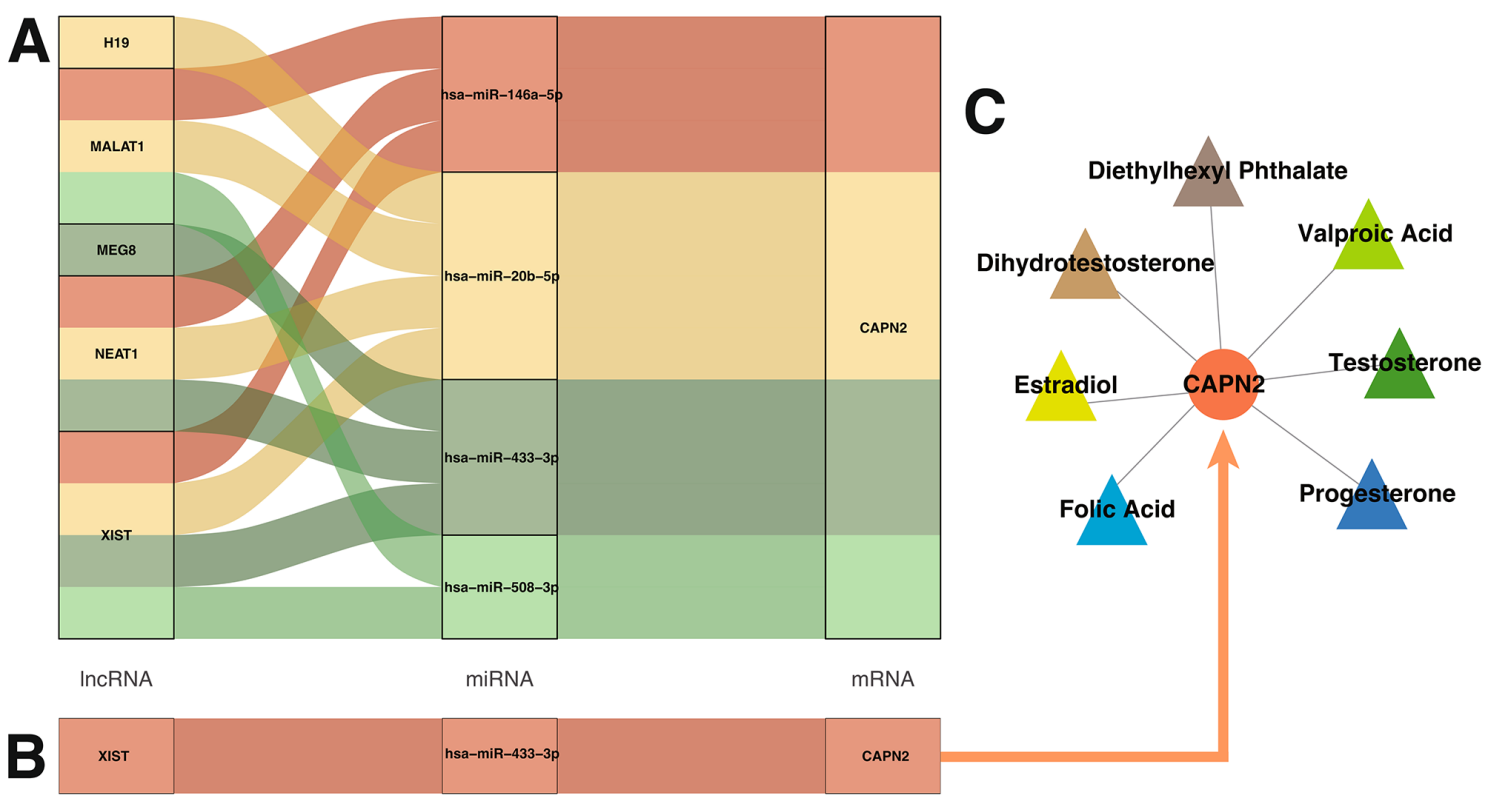
ceRNA Network and CTD Drug Predictions. (**A**) Outlines a ceRNA network with key differentially expressed miRNAs, lncRNAs, and CAPN2, detailing their regulatory interactions. (**B**) Condenses the network into a targeted ceRNA model that clarifies miRNA and lncRNA regulatory patterns with CAPN2, suggesting potential impacts on PCOS. (**C**) Utilizes the CTD to suggest drugs targeting CAPN2, guiding future treatment options based on the ceRNA network’s insights

Subsequently, the ENCORI database was employed to predict miRNAs targeting the CAPN2 gene, resulting in 127 potential mRNA-miRNA pairs. A subset of these miRNAs, specifically intersecting with the differentially expressed miRNAs from GSE138572, yielded four miRNAs: miR-146a-5p, miR-20b-5p, miR-433-3p, and miR-508-3p. Further database searches identified lncRNAs corresponding to these four miRNAs. An intersection with the differentially expressed lncRNAs from GSE138518 revealed five lncRNAs: H19, MALAT1, MEG8, NEAT1, and XIST (Fig. 4A).

Considering the mechanisms of ceRNA interactions, an inverse relationship is expected between the expression trends of target genes and lncRNAs, in relation to miRNAs. This led to the discovery of a potential ceRNA network, specifically XIST acting through hsa-miR-433-3p to regulate CAPN2 (Fig. 4B).

To identify drugs targeting the CAPN2 gene (Fig. 4C), we queried the CTD database with ‘CAPN2’ as a keyword, focusing on compounds associated with PCOS. The search identified seven drugs: Diethylhexyl Phthalate, Dihydrotestosterone, Estradiol, Folic Acid, Progesterone, Testosterone, and Valproic Acid, all relevant to endocrine regulation, particularly in reproductive health.

### Validation of the expression of CAPN2, XIST, and mir-433-3P in PCOS

Following informed consent, we recruited 12 patients with irregular menstrual cycles and more than 25 antral follicles, diagnosed with PCOS. Correspondingly, 12 patients with regular menstrual cycles and fewer than 15 antral follicles, seeking IVF-ET treatment due to tubal factors, were also enrolled. Table 1 summarizes the demographic baseline data of the 24 volunteers, showing that apart from a significant difference in BMI, other differences between the PCOS group and the control group were not pronounced. Notably, the PCOS patients exhibited higher levels of AMH, basal LH, and testosterone compared to the control group. After identical controlled ovarian stimulation in all volunteers, granulosa cells were collected on the day of oocyte retrieval. Total RNA extracted from these cells was analyzed using qPCR to validate the expression levels of CAPN2, XIST, and miR-433-3p.

**Table 1:** Comparative Clinical and Hormonal Characteristics of PCOS and Normal Subjects

	Normal *n* = 12	PCOS *n* = 12	*P*-value
Infertility Duration (years)	2.9 ± 1.7	4.3 ± 1.8	0.417
Age (years)	33.6 ± 3.8	28.3 ± 3	0.532
BMI	21.1 ± 2.9	23.5 ± 1.6	0.016
Age at Menarche	13	13	-
Menstrual Cycle Duration (days)	28–31	irregular	-
Basal FSH (mIU/mL)	6.6 ± 0.8	6.2 ± 0.7	0.444
Basal LH (mIU/mL)	6.1 ± 1.3	10.1 ± 0.7	0.108
Basal Prolactin (mIU/L)	345.8 ± 170.7	219.8 ± 254	0.238
Basal Estradiol (pg/mL)	102.2 ± 61.8	95.5 ± 55.8	0.994
Basal Progesterone (nmol/L)	1.1 ± 0.9	0.6 ± 0.4	0.265
Basal Testosterone (nmol/L)	0.8 ± 0.5	1 ± 0.6	0.612
AMH (ng/mL)	7.5 ± 1.6	12.9 ± 3.6	0.053
AFC	< 15	> 25	-
FSH on COS Initial Day (mIU/mL)	5.7 ± 1.9	6.2 ± 1.1	0.089
LH on COS Initial Day (mIU/mL)	4.9 ± 2.6	9.7 ± 3.3	0.758
Estradiol on COS Initial Day (E2) (pg/mL)	88.8 ± 68.3	94 ± 63	0.747
Initial Dosage of COS (IU)	158.3 ± 35.5	133.3 ± 17.1	0.038
COS Duration (days)	8.9 ± 1.4	9.3 ± 2.1	0.335
Gonadotropin Dosage within COS (IU)	1497.9 ± 472.6	1252.1 ± 391.7	0.38

COS: Controlled Ovarian Stimulation; FSH: Follicle Stimulating Hormone; LH: Luteinizing Hormone; AMH: Anti-Müllerian Hormone; AFC: Antral Follicle Count; Gn: Gonadotropin

The results of our study demonstrated a notable upregulation in the expression levels of CAPN2 and XIST in the PCOS group, suggesting a potential role in the pathophysiology of the syndrome. In contrast, the expression of miR-433-3p was significantly decreased in the PCOS group compared to the control group. This differential expression pattern may reflect the complex endocrine and metabolic dysregulations associated with PCOS, potentially offering insights into novel biomarkers or therapeutic targets.

**Fig. 5:**
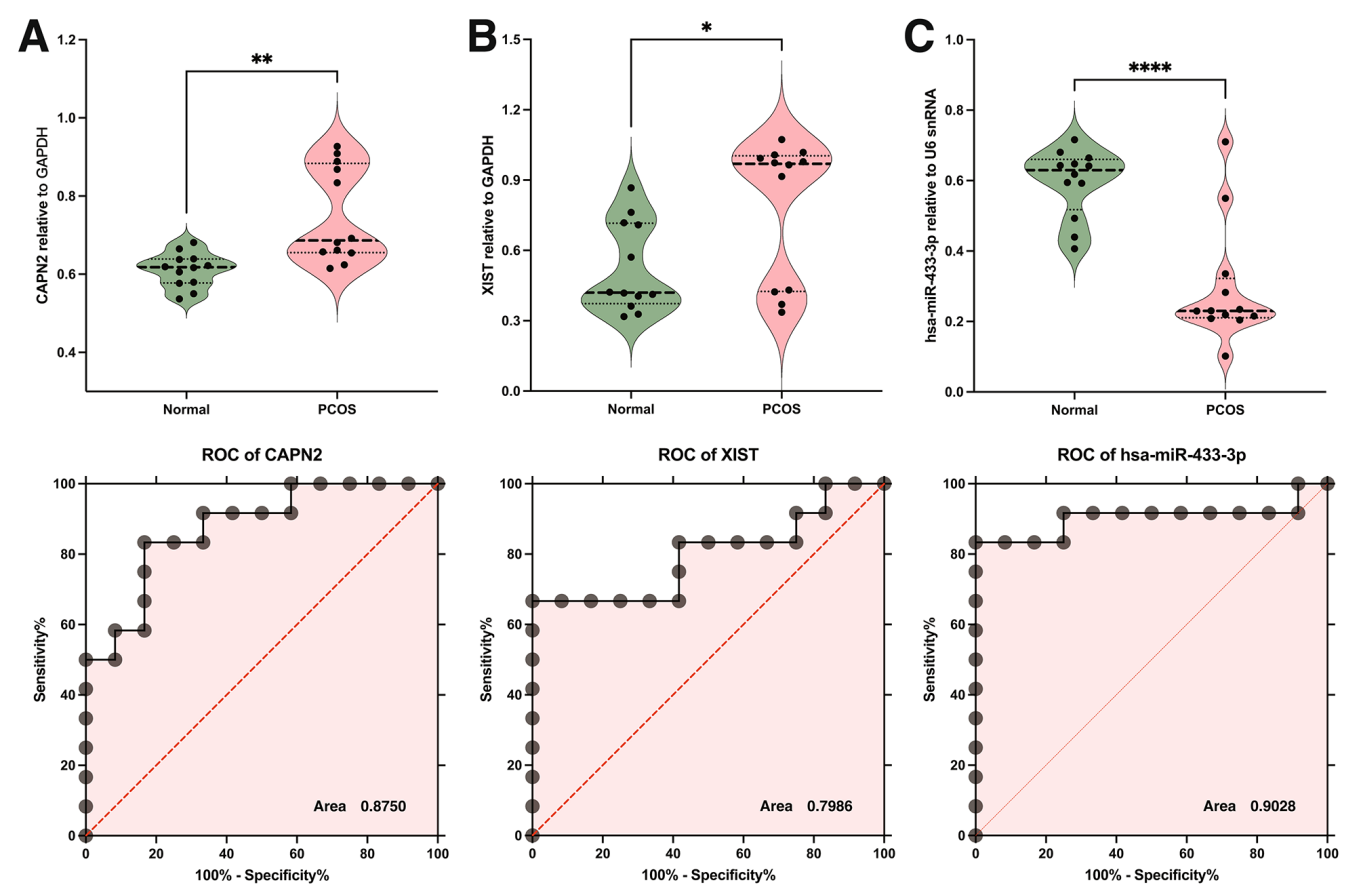
Expression and Diagnostic Analysis of CAPN2, XIST, and miR-433-3P in PCOS. (**A-C**) Upper panels: Violin plots illustrate the expression of CAPN2 (A), XIST (B), and hsa-miR-433-3p (C) in normal and PCOS samples, showing distribution and median expression levels. Lower panels: ROC curves evaluate the ability of these biomolecules to distinguish between PCOS and normal conditions, with AUC values indicating diagnostic accuracy. These analyses underscore the roles of these biomarkers in PCOS and their diagnostic relevance. * *P* < 0.05, ** *P* < 0.01, **** *P* < 0.0001

Furthermore, the efficacy of these molecular markers in distinguishing PCOS was rigorously evaluated using ROC analysis. The analysis revealed that CAPN2, XIST, and miR-433-3p collectively demonstrated substantial discriminatory power. Specifically, the AUC values ranged from 0.7986 to 0.9028, indicating a high level of accuracy in differentiating PCOS from normal ovarian function. This finding underscores the potential utility of these markers in clinical diagnostics. For a detailed illustration of these results, refer to Fig. 5, which graphically represents the ROC curves and highlights the sensitivity and specificity of each marker at various threshold levels.

This exploration of molecular markers in PCOS not only aids in understanding the underlying biological mechanisms but also paves the way for developing more precise diagnostic criteria and personalized treatment strategies. The significant variance in expression levels of these markers between PCOS and control groups warrants further investigation, potentially leading to breakthroughs in the management of this complex condition.

## Discussion

This study delves into the genetic and molecular mechanisms linking IR and PCOS, using a combination of bioinformatics tools and experimental validation to identify key genes associated with IR in PCOS patients. Key findings include the identification of thirteen genes correlated with IR, notably the upregulation of the CAPN2 gene, as confirmed by qPCR analysis. Additionally, we constructed a ceRNA network involving lncRNA XIST and hsa-miR-433-3p, linked to the CAPN2 gene. These findings not only shed light on the molecular regulatory mechanisms of ceRNA networks in PCOS-related IR but also provide potential targets for pharmacological intervention in treating this condition.

PCOS is a complex disorder characterized by hyperandrogenism, irregular ovulation, and polycystic ovaries, typically manifesting during puberty. Its etiology encompasses genetic predispositions, such as gene variants influencing steroidogenesis [28] and insulin action [29], the latter impacting glucose metabolism, as well as environmental factors such as lifestyle choices and prenatal toxin exposure [30]. The pathophysiology of PCOS is characterized by a dysfunctional hypothalamic-pituitary-ovarian axis [31], leading to menstrual irregularities and infertility. Hyperandrogenism in PCOS, presenting as acne and hirsutism, stems from excessive androgen production that disrupts ovarian follicle development [32]. Oxidative stress, a significant factor in PCOS, contributes to tissue damage, inflammation, and an increased risk of cardiovascular diseases and cancers [33]. Furthermore, AGEs in PCOS exacerbate reproductive and metabolic alterations by inducing inflammation and cellular damage [34].

Research into PCOS has advanced with efforts to identify biomarkers, notably through studies like Heidarzadehpilehrood et al., which used WGCNA to find novel lncRNAs linked to PCOS [35]. These lncRNAs, associated with key PCOS pathways such as gene expression and metabolism, were identified through gene expression analysis of patient and control samples. The study further identified crucial PCOS biomarkers and therapeutic targets by analyzing differentially expressed miRNAs [36]. Given the complexity of PCOS etiology, merely focusing on overarching biomarkers is insufficient. There has been a lack of research on the metabolic abnormalities’ mechanisms affected by PCOS. Our work zeroes in on the specific metabolic mechanisms and insulin resistance in PCOS, aiming to pinpoint precise therapeutic targets. This targeted approach intends to improve treatment effectiveness by addressing the intricacies of metabolic dysfunction.

IR, a critical aspect of PCOS, is characterized by a diminished biological response to insulin, adversely affecting glucose transfer and utilization. Regardless of body weight, a significant number of women with PCOS exhibit insulin resistance, highlighting its prevalence across various body types [37]. This condition, not exclusively linked to obesity, is also prevalent in lean individuals with PCOS, underscoring its complexity. Insulin plays a crucial role in maintaining homeostasis, lipid synthesis, and influencing steroidogenesis in the ovaries and adrenal cortex [38]. IR leads to hyperinsulinemia, which in turn causes excessive androgen secretion and reduced SHBG synthesis, thereby increasing testosterone levels [39]. Particularly during puberty, the early onset of IR and hyperinsulinemia can contribute to the development of PCOS phenotypes. Consequently, women with PCOS and IR face an elevated risk of developing diabetes and cardiovascular diseases, highlighting the importance of early intervention and management [40].

IR is a multifactorial condition shaped by a complex interplay of genetic, metabolic, epigenetic, and regulatory mechanisms, each contributing to its pathogenesis and progression. it involves complex disruptions in signaling pathways, prominently featuring genes like INSR, IRS1, IRS2, PI3K, Akt, and GLUT4 [41, 42]. The cascade initiated by lipid metabolites like diacylglycerol activates kinase pathways, impairing the insulin signal transduction. This disruption is notable in the PI3K/Akt/GLUT4 pathway, crucial for glucose uptake and metabolism in skeletal muscle and adipose tissues, highlighting the pathway’s central role in maintaining insulin sensitivity [43].

Beyond the signaling pathways, the metabolic dimension of IR involves crucial genes and mediators like GLUT4, adiponectin, and chemerin. These genes and adipokines regulate glucose and lipid homeostasis [44]. Dysregulation in lipid metabolism genes and adipokines contributes significantly to the development of IR, underlining the vital role of adipose tissue and its secreted factors in modulating the body’s metabolic profile and insulin sensitivity [45].

Besides, DNA methylation and histone modifications in key insulin signaling and mitochondrial genes, including INS, IGF-1/2, IGFBP-1/2, PPARG, and PPARA, significantly influence the development of IR [46]. Furthermore, non-coding RNAs, particularly miRNAs like miR-375, miR-150, miR-30a-5p, and miR-15a, play a pivotal role in regulating these epigenetic changes and mitochondrial functions, further complexifying the pathogenesis of IR [47, 48]. These miRNAs modulate gene expression and epigenetic landscapes, impacting insulin signaling, metabolic pathways, and mitochondrial health, thereby influencing the overall insulin responsiveness of tissues.

The CAPN gene family, which includes the CAPN2 gene, plays a crucial role in various cellular processes. CAPN2 is not only essential in protein degradation and modification, vital for maintaining cellular protein homeostasis [49, 50], but also significantly contributes to cell signaling, affecting cell proliferation, differentiation, and apoptosis [51–53]. Additionally, CAPN2 is involved in cytoskeletal regulation, which influences cell migration and morphology [54], and it participates in cell cycle regulation, especially in mitosis [55]. Within this gene family, CAPN10 has emerged as a novel factor in insulin resistance [56]. Its role in insulin signaling pathways is particularly important in the development of type 2 diabetes, as it impacts the efficiency of insulin receptor signaling and glucose uptake, thereby modulating the body’s insulin sensitivity [57]. Our research introduces the concept of a significant correlation between CAPN2 and insulin resistance, akin to that observed with CAPN10. However, whether the mechanisms underlying the role of CAPN2 in insulin resistance are identical to those of CAPN10 warrants further in-depth investigation.

XIST is essential in X-chromosome inactivation, crucial for dosage compensation in female mammals by silencing one of the X chromosomes [58]. On the other hand, miR-433-3p, a microRNA, is implicated in post-transcriptional gene regulation, affecting various cellular functions such as proliferation, differentiation, and apoptosis [59]. In our study, predictions were made using the ENCORI database and were validated effectively in datasets GSE138518 and GSE138572, as well as in our collected PCOS samples. Although significant correlations were found between XIST, CAPN2, and miR-433-3p with IR-related PCOS, the regulatory patterns of these molecules require further validation through more in-depth molecular experiments.

Further research could enhance understanding by expanding genetic analyses, conducting clinical trials for novel treatments, and considering diverse patient populations. A deeper exploration into molecular mechanisms and pathways, coupled with the integration of various omics data, would provide a more comprehensive view of the disease. Additionally, adopting patient-centered approaches would aid in developing personalized treatment strategies, potentially improving outcomes for those suffering from PCOS and insulin resistance.

## Conclusions

This study has successfully identified thirteen genes associated with IR in PCOS, highlighting the upregulation of the CAPN2 gene as a notable finding. Through the application of various bioinformatics tools and experimental validations, we have established a potential ceRNA network. This network, involving lncRNA XIST and hsa-miR-433-3p, suggests a regulatory linkage with CAPN2, a crucial element in the pathophysiology of PCOS. Additionally, our drug prediction analysis has identified seven pharmacological agents, mainly regulators of the endocrine system, offering new avenues for therapeutic intervention to address insulin resistance in PCOS patients. These findings provide a deeper understanding of the genetic and molecular underpinnings of PCOS and open up possibilities for the development of targeted treatment strategies, potentially improving the management and outcomes of this complex condition.

### Electronic supplementary material

Below is the link to the electronic supplementary material.

Supplementary Material 1

Supplementary Material 2

## Data Availability

No datasets were generated or analysed during the current study.

## Abbreviations

PCOS: Polycystic Ovary Syndrome
GEO: Gene Expression Omnibus
IRGs: Insulin Resistance-related Genes
MSigDB: Molecular Signatures Database
WGCNA: Weighted Gene Co-expression Network Analysis
IR: Insulin Resistance
LASSO: Least-Absolute Shrinkage and Selection Operator
SVM: Support Vector Machine
qPCR: Quantitative Polymerase Chain Reaction
DEGs: Differentially Expressed Genes
DE-lncRNAs: Differentially Expressed Long Non-coding RNAs
DE-miRNAs: Differentially Expressed MicroRNAs
ceRNA: Competing Endogenous RNA
MAD: Median Absolute Deviation
CTD: Comparative Toxicogenomics Database
ssGSEA: Single-sample Gene Set Enrichment Analysis
RFE: Recursive Feature Elimination
hsa: Human Sequence Archive (as in hsa-miRNA-433-3p)
CAPN2: Calpain 2
IVF-ET: In Vitro Fertilization-Embryo Transfer
cDNA: Complementary DNA
AGEs: Advanced Glycation End Products
