# Supplementary material for: CAPN2 correlates with insulin resistance states in PCOS as evidenced by multi-dataset analysis
Source: J Ovarian Res. 2024 Apr 12;17:79. doi: 10.1186/s13048-024-01407-2 (PMC11015649; doi:10.1186/s13048-024-01407-2)
Supplement: Supplementary file 1 — Supplementary Material 1 [file 13048_2024_1407_MOESM1_ESM.docx]

**Supplementary Table 1. Primers of qPCR.**

| CAPN2 | Forward | GTTCTGGCAATACGGCGAGT |
| --- | --- | --- |
|  | Reverse | CTTCGGCTGAATGCACAAAGA |
| GAPDH | Forward | TGTGGGCATCAATGGATTTGG |
|  | Reverse | ACACCATGTATTCCGGGTCAAT |
| XIST | Forward | GTAGGTGTGCTGATAACCAAGGC |
|  | Reverse | GGGAAAGGAAGATTGAGGGTGG |
| hsa-U6 | hsa-U6 qPCR Primer kit, CD201-0145, TIANGEN, Beijing, China | |
| hsa-miRNA-433-3p | hsa-miR-433-3p qPCR Primer kit, CD201-0478, TIANGEN, Beijing, China | |
